# Clinical Spectrum and Treatment Approaches in Corneal Burns

**DOI:** 10.4274/tjo.99267

**Published:** 2015-10-05

**Authors:** İlkay Kılıç Müftüoğlu, Yonca Aydın Akova, Altuğ Çetinkaya

**Affiliations:** 1 İstanbul Training and Research Hospital, Clinic of Ophthalmology, İstanbul, Turkey; 2 Bayındır Hospital, Clinic of Ophthalmology, Ankara, Turkey; 3 Dünya Eye Hospital, Ankara, Turkey

**Keywords:** Ocular surface, alkali burn, medical treatment, surgical treatment

## Abstract

**Objectives:**

To evaluate the clinical findings, treatment modalities and long-term prognosis of chemical and thermal burns of the cornea.

**Materials and Methods:**

Twenty-one patients (27 eyes) who were followed at two centers for corneal chemical and thermal burns between 2001 and 2013 were included. Eyes were grouped into four grades according to the severity of burn using Roper-Hall classification. Age, gender, type of burn, follow-up duration, corrected visual acuity before and after treatment, treatment modalities and complications were recorded. Patients received medical treatment or combined surgical treatment including amniotic membrane transplantation (AMT), conjunctivolimbal autograft/allograft (CLAU/CLAL) transplantation, keratolimbal allograft (KLAL) or penetrating keratoplasty (PKP).

**Results:**

Patients had a mean age of 27.1±15.5 years (range, 6 months-56 years) and were followed for a mean 63.2±58.6 weeks (4-160 weeks). Significant improvement was achieved with medical treatment alone in patients with grade I (4 eyes) and 2 burns (8 eyes). Patients with grade III burns (11 eyes) underwent CLAU (6 eyes), combined AMT/CLAU (3 eyes), AMT/CLAL (1 eye), or CLAL+PKP (1 eye), while patients with grade IV burns (4 eyes) had keratectomy+CLAL/AMT (1 eye), keratectomy+CLAL+PKP after recurrence with CLAU/AMT (1 eye), CLAU+PKP (1 eye), and AMT/KLAL+PKP (1 eye). All patients except the latter showed ocular surface stabilization with these procedures.

**Conclusion:**

Ocular burns cause severe impairment of the ocular surface. It is possible to achieve good results with appropriate medical treatment and surgeries including ocular surface reconstruction.

## INTRODUCTION

Chemical and thermal corneal burns lead to rapid and progressive destruction and serious anterior segment complications.^1^ Chemical burns comprise 12% of all eye trauma cases. The severity of resulting complications is affected by the concentration of the chemical agent, the pH, duration of exposure, and extent of contact with the ocular surface and tissues.^1,2^

Ocular chemical burns are the most common cause of limbal stem cell deficiency and are characterized by non-healing epithelial defects, stromal inflammation, neovascularization, conjunctivalization and corneal opacification.^1^

In the treatment of chemical burns, clearing the agent from the ocular surface and suppressing inflammation are essential in the acute phase, whereas managing complications and ensuring a healthy ocular surface are important in the chronic phase.

In recent years, a combination of appropriate medical treatment in the acute phase and ocular surface reconstruction has begun to yield desired results in cases of chemical ocular injury.^3,4,5,6,7,8,9^

In this study, we aimed to present the treatment methods used, responses to treatment and long-term prognoses in cases we have followed due to corneal burns.

## MATERIALS AND METHODS

The study included 27 eyes of 21 patients who were followed and treated by the same physician (Y.A.A.) for chemical or thermal burns between 2001 and 2013 at the Başkent University Medical School Department of Ophthalmology and later at the Bayındır Hospital Eye Clinic. Patients’ data were screened retrospectively; age, gender, causative agent, duration of follow-up, time elapsed between exposure and presentation to the hospital, best corrected visual acuity (BCVA) at time of presentation, treatment modalities applied, post-treatment BCVA and complications were recorded. For statistical analysis, BCVA values expressed as decimals were converted to logMAR.10 Counting fingers was evaluated as logMAR 2.00, hand motion as logMAR 3.00.

Patients’ limbal function was assessed clinically by slit-lamp examination. Limbal dysfunction was evaluated as findings of advancement of the conjunctival epithelium onto the cornea, loss of limbal Palisade of Vogt and/or peripheral surface neovascularization.

Based on corneal and conjunctival involvement, burn severity was graded according to the Roper-Hall classification system.^11^ In this classification, corneal epithelial defects but no conjunctival/corneal ischemia is grade I; corneal haze but visible iris details and less than one third limbal ischemia is grade II; total epithelium loss, stromal haze, obscured iris details and between one third to one half limbal ischemia is grade III; and opaque cornea, obscured iris and pupil details and more than one half limbal ischemia is grade IV.

The BCVA of grade I, II and III patients was evaluated as previously described; grade IV patients had very low BCVA, at the level of light perception; eyes in this group were not included in BCVA calculations and were evaluated separately.

For patients receiving medical treatment, topical corticosteroid drops, preservative-free artificial tears (hydroxypropyl methyl cellulose), autologous serum and topical vitamin A drops (Vitamin A-POS 250 IU/g, Biem İlaç San. ve Tic. A.Ş., Ankara, Turkey) were administered; for patients who developed progressive corneal neovascularization despite those treatments, topical bevacizumab (5 mg/ml, four times daily) drops were added after a period of 4-6 weeks. Corneal neovascularization was clinically evaluated using biomicroscopic digital images of the anterior segment taken during follow-up. As surgical treatment, amniotic membrane transplantation (AMT), conjunctival limbal autograft (CLAU) transplantation, conjunctival limbal allograft (CLAL) transplantation, keratolimbal allograft (KLAL) transplantation or penetrating keratoplasty (PKP) were performed individually, sequentially or in combination depending on the severity of the damage.

Procedures related to the ocular surface were performed by the same surgeon (Y.A.A.) under local or general anesthesia. Patients with unilateral limbal stem cell deficiency underwent CLAU transplantation. In this technique, surface keratectomy and conjunctival peritomy including the limbal area were performed initially to remove the vascularized pannus tissue, after which cautery was used to achieve adequate hemostasis. From the fellow healthy eye, limbal-conjunctival tissue 8 mm wide in the horizontal plane and 5-8 wide in the vertical plane was obtained, with care taken not to take Tenon’s or episcleral tissue. The graft tissue was placed and sutured in an anatomically correct position over the quadrant of the recipient bed with most severe limbal insufficiency. Postoperatively a bandage contact lens was installed to protect the ocular surface. For 3 patients, the CLAU procedure was performed with fibrin glue. In this technique, while one fixation suture is placed in the conjunctival surface to provide graft stabilization, no sutures are placed in the corneal surface.

Patients with bilateral limbal deficiency underwent CLAL transplantation and/or KLAL transplantation using a graft taken from an HLA-compatible living relative or a cadaveric KLAL. The procedure for CLAL transplantation was similar to that described for CLAU; KLAL transplantation was conducted using the 360° corneoscleral ring remaining after an 8 mm trephination, divided into two segments.

In the postoperative period, patients were treated four times daily with prednisolone acetate drops (Pred Forte, Allergan, Westport Co, Mayo, Ireland) or dexamethasone drops (Dexasine SE, Liba, İstanbul, Turkey); ofloxacin drops (Exocin, Allergan, Westport Co, Mayo, Ireland) or moxifloxacin drops (Vigamox, Alcon, FW, Texas, USA); and preservative-free artificial tears. Topical antibiotic was used until the corneal epithelium healed, while topical corticosteroid drops were gradually reduced according to clinical response. Two limbal allograft transplantation patients also received systemic cyclosporin (Sandimmun, Novartis Pharma, Basel, Switzerland) for a period of 2 years (5 mg/kg for the first 6 months, 3 mg/kg for the remaining 18 months). Systemic steroid (Deltacortil, Ultralan-Oral, Bayer Türk, İstanbul, Turkey) treatment was administered for 6 to 12 months (at a starting dose of 1 mg/kg/day and gradually decreasing). While patients were using immunosuppressive agents, they were monitored for possible systemic side effects by monthly biochemical testing.

Patients with eyelid dysfunction following the chemical burn underwent entropion correction, symblepharon lysis surgery and/or buccal mucosal graft surgery prior to limbal stem cell transplantation to prevent corneal exposure.

Increase in visual acuity, improvement in ocular surface stability, corneal transparency and complications were evaluated at the end of the follow-up period. A successful outcome was defined as an increase in visual acuity of at least one row, whereas cases exhibiting persistent epithelial defect lasting longer than two weeks, progressive corneal vascularization and/or corneal thinning were considered unsuccessful. In patients who underwent penetrating keratoplasty, presence of persistent corneal epithelial disorders and vascularization and opacity in the 4 mm central area was accepted as an unsuccessful outcome.

SPSS version 18.0 for Windows (SPSS, Chicago, IL, USA) was used for statistical analyses. Pre- and post-treatment values were compared using the Wilcoxon test. Level of significance was accepted as α=0.05.

## RESULTS

The study included 27 eyes of 21 patients (14 male, 7 female) with an average age of 27.1±15.5 years (range, 6 months-56 years). Patients were followed for an average of 63.2±58.6 weeks (range, 4-160 weeks). Three patients (14.3%) had thermal burns, while 1 patient (4.8%) had an acid chemical burn and 17 patients (80.9%) had alkali chemical burns. Three patients (14.3%) presented to our clinic immediately following exposure and 1 (4.8%) presented within one week. The remaining patients (80.9%) presented at our clinic an average of 46.2±81.3 weeks (range, 12-78 weeks) after exposure.

Injury severity was classified based on corneal-conjunctival involvement and limbal ischemia as follows: grade I for 4 eyes (14.8%), grade II for 8 (29.7%), grade III for 11 (40.7%), and grade IV for 4 (14.8%).

The average BCVA of grade I patients was 0.2±0.2 (0.5-0 logMAR) at initial examination and 0.05±0.05 (0.1-0 logMAR) after treatment. BCVA values for grade II and III patients before treatment were 1.2±1.13 (3-0.4 logMAR) and 0.26±0.19 (0.7-0.1 logMAR), respectively, and, 0.45±0.3 (0.9-0.2 logMAR) and 0.18±0.27 (0.7-0 logMAR) respectively, at the final examination. These increases in visual acuity were statistically significant (p<0.05). Of the 4 patients with grade IV burns, BCVA increased from light perception to counting fingers at 1 meter for one patient, from light perception to 0.2 (Snellen) for one patient, and from hand movement to 0.05 (Snellen) in one patient. Despite treatment, the vision of the other grade IV patient deteriorated from light perception to light perception negative due to total vascularized leukoma.

**Figure 1:**
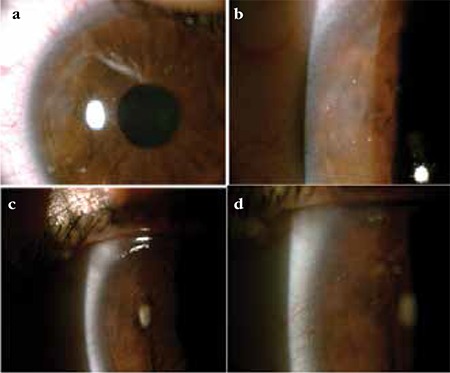
Patient with grade II burn. a, b) Pre-treatment anterior segment photographs, c, d) Post-treatment anterior segment photographs. Peripheral corneal vascularization has decreased and corneal opacity has disappeared with treatment.

**Figure 2:**
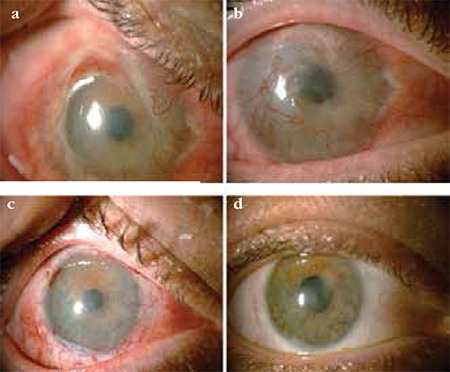
Patient with grade III burn. a, b) Pre-treatment images from a patient with grade III burn. c) The same patient 1 month after limbal autograft and amniotic membrane transplantation. d) The same patient at 3 months after treatment.

Significant improvement and stabilization of the ocular surface was achieved in grade I and II patients with medical treatment alone (Figure 1a, 1b, 1c, 1d). Among grade III and IV patients, 2 (13.3%) underwent a single surgical procedure (CLAU); 13 (86.6%) underwent combined or sequential procedures. Surgical procedures for grade III patients were as follows: CLAU (6 eyes); AMT/CLAU (3 eyes); AMT/CLAL (1 eye); and CLAL followed by PKP (1 eye). For grade IV patients: keratectomy, AMT/CLAL (1 eye); keratectomy, AMT/CLAU and subsequently keratectomy, CLAL and PKP due to recurrence (1 eye); keratectomy and repeated CLAU, AMT/KLAL followed by PKP (1 eye) (Figure 2a, 2b, 2c, 2d). PKP was performed an average of 9.3±7.6 months (range, 8-23 months) after limbal stem cell transplantation. Three patients underwent a single PKP; one patient underwent PKP twice.

**Figure 3:**
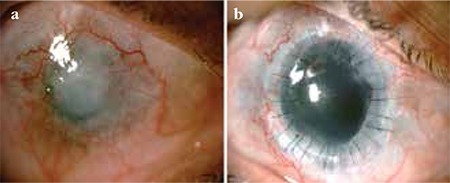
Patient with grade IV burn. a) Pre-treatment condition of a patient with grade IV burn. b) The same patient 6 months after keratolimbal allograft transplantation and penetrating keratoplasty

In 2 of the grade IV patients, limbal stem cell deficiency recurred after CLAU in one and after KLAL and PKP in the other. In the first patient, a relatively stable ocular surface was achieved and BCVA increased with KLAL and PKP (Figure 3a, 3b). However, in the second case, even after repeated KLAL and PKP, ocular surface stability could not be achieved due to the development of vascularized leukoma.

Besides the surgical procedures to the ocular surface, entropion surgery was performed prior to CLAU or CLAL in 2 eyes with trichiasis and entropion; buccal mucosal membrane graft with eyelid and fornix reconstruction was performed in 2 eyes with symblepharon. Procedures related to the ocular surface were performed at least 6 months after eyelid function returned to normal.

During the follow-up period, 2 eyes (7.4%) developed glaucoma and 2 eyes (7.4%) formed cataracts. Intraocular pressure was controlled in one glaucoma patient with topical antiglaucoma treatment; in the other patient, glaucoma developed in month 37 and was controlled by Ahmed glaucoma valve implantation. Cataract surgery was not performed on one patient because there was no significant impact on visual acuity; the second patient underwent phacoemulsification and toric intraocular implantation with a resulting postoperative BCVA of 0.2 logMAR.

## DISCUSSION

Corneal burns can lead to serious complications due to ocular surface damage. Today, it is possible to limit these complications with current medical treatment options and advanced surgical techniques.

The severity of ocular surface damage depends on the agent type, pH and length of exposure. The majority (80.9%) of corneal burns in our case series were caused by alkali agents, and 48.1% of patients with alkali damage were in a higher severity grade (grade III-IV). Having a lipophilic structure, alkaline substances penetrate the eye and damage the ocular surface, ciliary body, trabecular meshwork and lens faster than acidic substances. The acute saponification of hydroxyl ions, cell membrane destruction with protein hydrolysis and denaturation are responsible for the pathogenesis of this damage.^1,2^ In cases of alkali burn, the inflammatory response rapidly progresses due to the release of proteolytic enzymes from damaged tissue, leading to limbal and conjunctival ischemia. This in turn leads to persistent corneal epithelial defects, trophic ulcers, corneal neovascularization and even corneal perforation. With acid burns, coagulative necrosis of the epithelium prevents damage from penetrating to deep tissues.^1,2^

After exposure to chemical agents, patients with lower stage injuries have good prognosis with medical treatment; however, patients with advanced stage burns require an ocular surface reconstructive procedure or a combination of multiple procedures in addition to medical treatment. In our grade I and II patients, ocular surface stability as well as significant increases in visual acuity were achieved with medical treatment alone. Some patients in these grades also received autologous serum in addition to standard medical treatment, as we believed it may contribute to epithelialization. We observed complete epithelialization in all of these patients. However, as this was not a controlled, comparative study and patients received combined treatment, it is difficult to assess the contribution of autologous serum to epithelialization. We believe the lysozymes, lactoferrin, epidermal growth factor and tumor growth factor in autologous serum may contribute to epithelialization. To clearly demonstrate this effect, controlled comparative studies with large patient numbers are needed.

In the treatment of corneal burns, AMT is used in the acute or chronic phase to help protect the conjunctival surface and reduce limbal stromal inflammation.^12,13,14,15^ AMT is used during ocular surface reconstruction to heal epithelial defects as well as provide pain control.^13,14,15^

Meller et al.^12^ performed AMT following conventional medical treatment in 13 eyes of 11 patients; they reported that AMT resulted in complete epithelialization, with limbal stem cell deficiency only developing in patients with grade IV burns. Tejwani et al.^13^ followed patients with alkali burns and reported epithelial defect healing in 92.9%, improvement of symptoms in 84.6%, and successful ocular surface reconstruction in 63.5% of patients.

The advantages of AMT in ocular surface reconstruction include reducing perilimbal inflammation, creating a healthy corneal epithelium, reducing corneal neovascularization and enhancing the success of subsequent limbal stem cell transplant and/or PKP.^2,16^ We used this technique primarily in grade III-IV patients in combination with CLAU or KLAL transplantation to provide a healthy ocular surface and/or prior to PKP to increase the likelihood of success. In addition to these cases, we also performed AMT to treat persistent epithelial defect in a 6-month old grade II patient who presented in the acute phase, and we observed complete corneal epithelialization in postoperative week 2. In a previous study, we performed AMT in combination with CLAU and symblepharon lysis in two pediatric patients and achieved a significant increase in vision level as well as ocular surface healing.^17^ There are limited data in the literature regarding the use of limbal stem cell transplantation in pediatric patients following chemical burns.^17,18^ Examination of pediatric patients may need to be done under general anesthesia, as slit-lamp examination can be difficult.

While satisfactory results are achievable in unilateral limbal stem cell deficiency with CLAU transplantation, the same is not true for patients with bilateral disease.^19,20,21,22^ For bilateral cases, heterologous tissue from cadavers or HLA-compatible living relatives may be used. A 5 to 6 clock hour sample of limbal tissue can be taken from the eye of a living donor without causing damage to the donor eye. However, it is possible to take larger amounts of tissue from cadavers.^21,23,24^ In addition to these options, the production of limbal stem cells in laboratory conditions has gained attention in recent years.^25^ This technique facilitates access to stem cells, and as it requires advanced equipment and experience, its use is expected to increase in coming years.

In a study comparing the outcomes of limbal stem cell transplantation using autografts and allografts, better corneal epithelialization and transparency were observed in the autograft group.^26^ Only one third of patients who underwent CLAU transplantation had corneal epithelialization after the first procedure; most eyes required repeated surgery. These results indicate that the corneal epithelium resulting from KLAL transplantation is more fragile than from CLAU transplantation.^26^ An advantage of autograft limbal stem cell transplantation over KLAL is that immunosuppression is not necessary; however, it must be kept in mind that these patients’ healthy eyes may develop limbal deficiency, especially after a trauma or possibly postoperatively.

In the literature, there are also data available regarding the use of combined CLAU/KLAL transplantation in cases of severe unilateral ocular surface failure.^27 Chan et al.27^ followed patients with preoperative 20/400 BCVA or worse for approximately 36 months and found postoperative BCVA of 20/80 or better in 73% and ocular surface stability in 82% of cases. In that study, 91% required additional PKP, and a success rate of 60% was reported.

For some of the CLAU transplantation patients in our study, fibrin tissue glue was used, which resulted in shorter surgery duration than the sutured procedure and significantly increased patient comfort. It is claimed that the use of fibrin glue also decreases the risk of postoperative wound infection due to its prevention of debris accumulation in the wound and the antibiotic effect of aprotinin, one of its ingredients. Due to its high cost, however, its use may be limited to selected patients with severe ocular surface disorders.

In some patients who underwent CLAU/CLAL to ensure the stability of the ocular surface, additional PKP surgery may be necessary due to serious postoperative corneal scarring. In our study, 4 patients required keratoplasty; these patients (1 with grade III and 3 with grade IV burns) underwent PKP in addition to limbal stem cell transplantation. In these cases, in order to place the corneal graft on a relatively non-inflamed corneal surface and decrease the risk of corneal graft rejection as much as possible, the two procedures were done at different times. It has also been demonstrated by researchers that fewer complications occur when PKP is performed years after limbal stem cell transplantation versus simultaneously.^28^ In our study, there was an average interval of 9.3±27.4 months (range, 8-23 months) between CLAU/KLAL transplantation and PKP. Of those 4 patients, 3 showed acceptable increases in visual acuity and relatively stable ocular surface, while in 1 patient graft decompensation occurred despite repeated KLAL and PKP.

With conjunctival limbal stem cell transplantation, the potential risk of limbal stem cell deficiency in the healthy eye is a concern. However, leaving the limbal stem cells in an area of 6 clock hours minimizes this risk. We were also careful to follow this guideline, and limbal deficiency did not occur in any of the patients’ healthy eyes or relatives’ donor eyes.

In KLAL transplantation, in contrast to PKP, the limbal tissue is placed directly on the recipient’s vascular bed instead of the avascular bed. Due to the Langerhans cells and antigenic material in limbal tissue, systemic immunosuppressive therapy is required in order to minimize graft rejection. Patients in our study received systemic cyclosporin A treatment for at least 2 years with monthly monitoring of biochemical parameters, as well as short-term oral steroid treatment. No serious immunosuppression-related side effects were observed in any of the patients during the follow-up period.

In corneal burn cases, it is important to manage complications as well as stabilize the ocular surface. One of the most significant complications is glaucoma. In the pathogenesis of glaucoma’s acute phase, stromal penetration of the agent results in collagen shrinkage in keratocytes and the stroma due to proteoglycan loss.^29^ Damage to the trabecular meshwork itself and the accumulation of inflammatory debris there are responsible for the chronic phase of glaucoma.^29^ While elevated intraocular pressure was not observed in our patients during the acute phase, glaucoma developed in the chronic phase in 2 cases, and was controlled with topical antiglaucoma medication (1 patient) and surgery (1 patient).

There are some limitations to this study. The patient sample size was small and the treatment protocols used were not evaluated comparatively. More conclusive results may be obtained by conducting a prospective, comparative evaluation of the treatment modalities with long follow-up periods.

In conclusion, chemical burns can cause ocular damage ranging from mild to severe. It is of utmost importance to ensure ocular surface healing and stabilization and manage complications during patient follow-up. Following each intervention, these patients should be monitored closely and physicians must be aware that repeated surgical procedures may be necessary.
